# Fundus camera and artificial intelligence for diabetic retinopathy screening in a São Paulo shelter

**DOI:** 10.1590/1806-9282.20260862

**Published:** 2026-10-16

**Authors:** Julia Gimenes da Silva Dias, Gabriel Costa de Andrade, João Victor Tenório Lossio de Macedo, Andrezza Soldera Graziano

**Affiliations:** 1 Irmandade da Santa Casa de Misericórdia de São Paulo São Paulo SP Brazil Irmandade da Santa Casa de Misericórdia de São Paulo – São Paulo (SP), Brazil.; 2 Irmandade da Santa Casa de Misericórdia de São Paulo Department of Ophthalmology São Paulo SP Brazil Irmandade da Santa Casa de Misericórdia de São Paulo, Department of Ophthalmology – São Paulo (SP), Brazil.

**Keywords:** Diabetic retinopathy, Photography, Screening, Artificial intelligence, Telemedicine

## Abstract

**OBJECTIVE:**

The aim of this study was to evaluate the applicability of a smartphone-based portable fundus camera for diabetic retinopathy screening in elderly residents of a specialized shelter in São Paulo, Brazil, considering image acquisition by non-ophthalmologist professionals and remote case classification.

**METHODS:**

Prospective pilot interventional study at the Centro de Acolhida Especial para Idosos Morada São João. Thirty-eight participants (76 eyes) underwent retinal imaging using the Eyer^®^ portable fundus camera (Phelcom Technologies, Brazil) by trained non-ophthalmologist professionals. Images were remotely evaluated by a retinal specialist and compared to the EyerMaps^®^ artificial intelligence system.

**RESULTS:**

Mean age was 71.6±7.0 years; 78.9% male. Fifteen participants (39.5%) had confirmed diabetes mellitus. The specialist identified abnormalities in 70 of 76 eyes (92.1%), most frequently glaucoma suspicion, media opacity suggestive of cataract, chorioretinal changes, and age-related macular degeneration signs; no diabetic retinopathy was identified. Against the specialist's assessment of any ocular abnormality as the reference standard, the artificial intelligence system classified 22 eyes as abnormal, yielding sensitivity 25.7% (95%CI 16.9–37.0), specificity 33.3% (95%CI 9.7–70.0), positive predictive value 81.8% (95%CI 61.5–92.7), negative predictive value 3.7% (95%CI 1.0–12.5), and overall accuracy 26.3% (95%CI 17.7–37.2).

**CONCLUSION:**

The Eyer^®^ device demonstrated operational feasibility for ocular screening by trained non-ophthalmologists. The high prevalence of media opacities and non-diabetic retinopathy conditions compromised artificial intelligence performance, highlighting the need for broader screening strategies in elderly and socially vulnerable populations.

## INTRODUCTION

Diabetes mellitus is one of the most significant public health challenges of our time, with chronic complications directly impacting morbidity, quality of life, and healthcare costs worldwide. Among these, diabetic retinopathy stands out as a leading cause of preventable blindness, affecting over 100 million people globally^1,2^.

In Latin America, prevalence ranges from 15.0% in Costa Rica to 32.7% in Brazil—the highest among countries assessed—reflecting disparities in diabetes prevalence and healthcare system inequalities^3^. In Brazil, this is compounded by the uneven distribution of ophthalmological services concentrated in urban referral centers, with limited primary care screening capacity.

Diabetic retinopathy is a progressive retinal complication of chronic hyperglycemia, classified as non-proliferative (mild, moderate, and severe) and proliferative, with diabetic macular edema possible at any stage. Early stages feature microaneurysms, hemorrhages, and hard exudates; advanced ischemia leads to neovascularization, vitreous hemorrhage, and tractional retinal detachment^4,5^. Despite this severity, visual loss is preventable with adequate screening and timely treatment—yet many patients remain asymptomatic until advanced disease^5-7^.

Portable fundus cameras and smartphone-based devices enable retinal imaging outside traditional settings, including primary care units and long-term care facilities (LTCFs) for the elderly, and artificial intelligence (AI) systems may reduce dependence on specialist evaluation^6-8^. Barriers are especially pronounced in elderly and vulnerable populations—functional limitations, comorbidities, and transportation difficulties contribute to underdiagnosis. On-site retinal imaging may overcome these barriers^3,6,7^.

This study evaluates the feasibility of the Eyer^®^ portable fundus camera (Phelcom Technologies, São Carlos, Brazil) for diabetic retinopathy screening in elderly residents of the Centro de Acolhida Especial para Idosos Morada São João, with images obtained by trained non-ophthalmologists. Secondarily, it investigates the diagnostic performance of EyerMaps^®^ AI compared to a retinal specialist.

## METHODS

### Study design and population

A prospective pilot interventional study was conducted at the Centro de Acolhida Especial para Idosos Morada São João, central São Paulo, Brazil, between April and September 2025. The study was approved by the Research Ethics Committee of Irmandade da Santa Casa de São Paulo on 30 January 2025 (Opinion No. 7.351.391; CAAE 85488024.7.0000.5479). All study procedures were performed after ethics approval: chart and demographic data were extracted from institutional records from 3 April 2025, and retinal image acquisition was carried out between July and September 2025. Participants were enrolled as a convenience series: all residents aged ≥18 years with a prior diagnosis of diabetes mellitus and/or systemic arterial hypertension—the latter included as a shared cardiovascular risk factor—who were present at the institution during the study period and agreed to sign the Informed Consent Form (ICF) were considered eligible. The aim was to assess imaging feasibility across residents at cardiometabolic risk rather than in diabetic residents exclusively. Individuals under 18 years or who did not consent were excluded. Of 209 residents present during the study period, 38 (18.2%) were enrolled. No a priori sample size calculation was performed given the exploratory nature of the study.

### Procedures and image acquisition

After informed consent, participants were assessed for demographic, clinical, and biochemical data from institutional records. Anterior segment and fundus images of both eyes were acquired using the Eyer^®^ device by non-ophthalmologist professionals (nursing staff, medical students, and physicians without prior ophthalmological experience), all trained by an ophthalmologist. All images were acquired without pharmacological mydriasis (non-mydriatic imaging). Images were uploaded to the EyerCloud^®^ cloud platform (Phelcom Technologies), compliant with the Brazilian General Data Protection Law (*Lei Geral de Proteção de Dados Pessoais* [LGPD]; Law No. 13,709/2018).

### Image evaluation and classification

A retinal specialist remotely classified images as gradable or non-gradable. For gradable images, diabetic retinopathy was categorized as non-referable (absent or mild-to-moderate non-proliferative without maculopathy) or referable (severe non-proliferative, proliferative, or diabetic macular edema). Non-gradable cases had anterior segment images reviewed for media opacities. The specialist performed the evaluation blinded to the EyerMaps^®^ output; conversely, the AI system processed images without access to participants’ clinical information. Specialist findings were subsequently compared to EyerMaps^®^ automated analysis.

### Statistical analysis

Categorical variables were presented as absolute and relative frequencies; continuous variables as mean±standard deviation or median (interquartile range). AI diagnostic performance was assessed by sensitivity, specificity, positive predictive value (PPV), negative predictive value (NPV), and overall accuracy, using the specialist's assessment as the reference standard. Ninety-five percent confidence intervals (95%CIs) for each metric were calculated using the Wilson score method.

## RESULTS

### Demographic and clinical characteristics

The flow of participants is illustrated in Figure 1. Thirty-eight participants (76 eyes) were included, predominantly male (78.9%), aged 60 to 90 years (mean 71.6±7.0). Fifteen (39.5%) had confirmed diabetes mellitus, 20 (52.6%) denied it, and 3 (7.9%) were unable to provide information. Table 1 summarizes the main sample characteristics.

**Figure 1:**
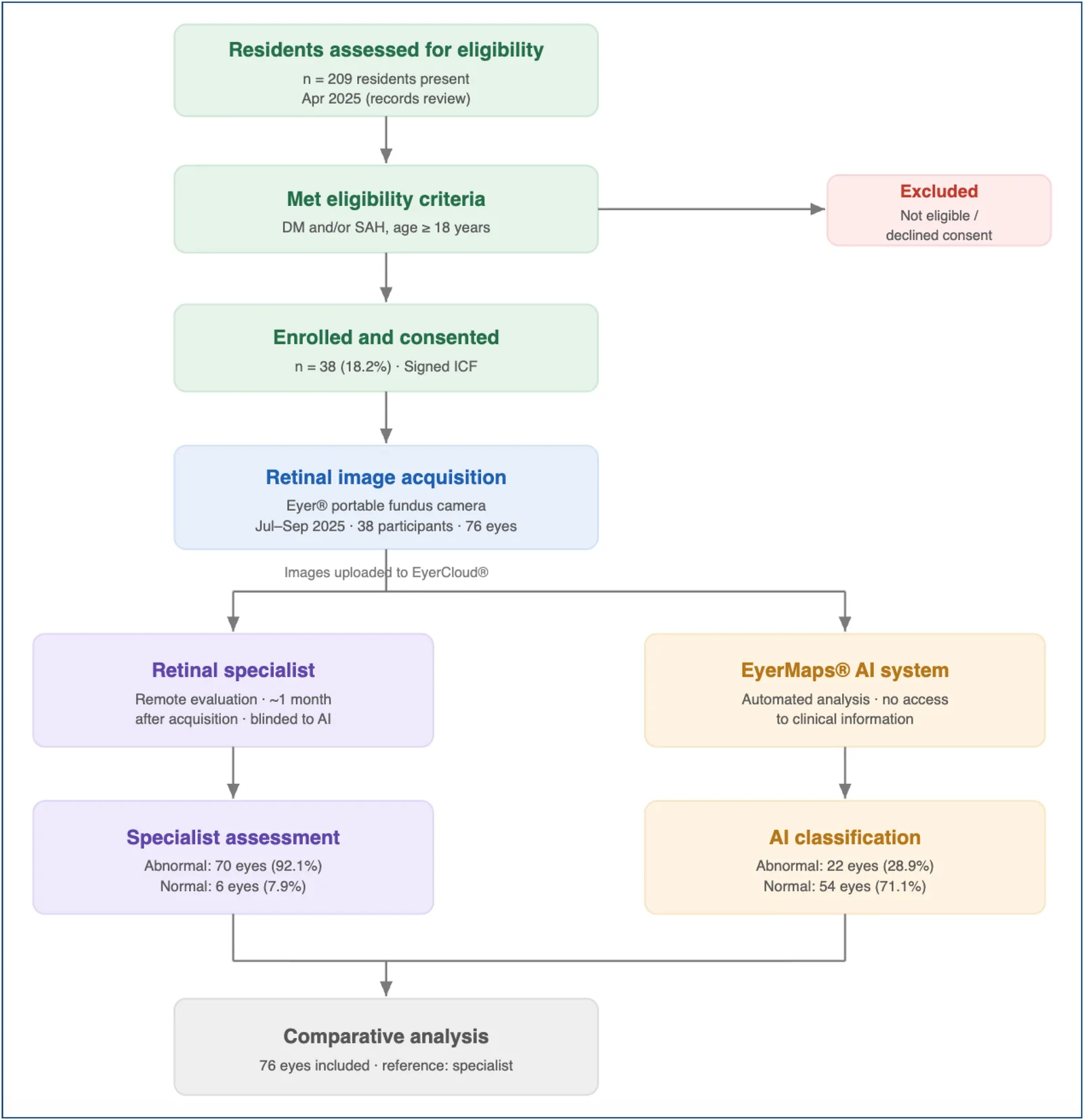
Flow of participants, from eligibility assessment to comparative analysis of specialist versus artificial intelligence classification. ICF: Informed Consent Form; DM: diabetes mellitus; SAH: systemic arterial hypertension; AI: artificial intelligence. [First author: Dias JGS—Figure 1].

**Table 1:** Demographic and clinical characteristics of participants (n=38).

Characteristic		n	%
Male sex		30	78.9
Age range (years)		60–90	–
Mean age (years)		71.6±7.0	–
Diabetes mellitus			
	Confirmed	15	39.5
	Denied	20	52.6
	Unknown	3	7.9
Comorbidities			
	Systemic arterial hypertension	30	78.9
	Dyslipidemia	12	31.6
	Previous cardiovascular disease	4	10.5
	Cataract	12	31.6
	Glaucoma (prior diagnosis)	3	7.9

All 38 medical records were reviewed; comorbidity data are complete, and absence of a listed comorbidity denotes documented absence of that diagnosis. Diabetes status was self-reported and could not be determined in three participants. HbA1c and diabetes duration were unavailable owing to the absence of digitized institutional records.

### Specialist findings

No adverse events were recorded during image acquisition. Ocular abnormalities were identified in 70 of 76 eyes (92.1%); only 6 eyes (7.9%) from two participants had no abnormal findings. No diabetic retinopathy (referable or non-referable) was identified among gradable eyes; 22 eyes were non-gradable owing to media opacity. Abnormalities were heterogeneous and predominantly unrelated to diabetic retinopathy: glaucoma suspicion or increased cup-to-disc ratio (31.6%), media opacity suggestive of cataract limiting fundus assessment (28.9%), tessellated and atrophic changes (13.2%), chorioretinal atrophy and scars (10.5%), age-related macular degeneration (AMD) signs (7.9%), disc pallor and peripapillary atrophy (10.5%), and tilted disc or pathological myopia (7.9%). Table 2 details the findings.

**Table 2:** Main categories of findings on fundus examination by the specialist (n=76 eyes).

Category of finding	Eyes (n=76)
No abnormalities	6 (7.9%)
Glaucoma suspicion/increased cup-to-disc ratio	24 (31.6%)
Media opacity/cataract (limiting assessment)	22 (28.9%)
Tessellated/senile atrophic changes	10 (13.2%)
Chorioretinal atrophy/scars	8 (10.5%)
AMD/drusen/RPE changes	6 (7.9%)
Disc pallor/peripapillary atrophy	8 (10.5%)
Tilted disc/pathological myopia	6 (7.9%)

AMD: age-related macular degeneration; RPE: retinal pigment epithelium. One eye may present multiple findings simultaneously.

### Artificial intelligence system performance

Retinal images were evaluated by the specialist approximately 1 month after acquisition. Using the specialist's assessment of any ocular abnormality as the reference standard, EyerMaps^®^ classified 22 eyes as abnormal and 54 as normal. Among the 70 eyes classified as abnormal by the specialist, the AI correctly flagged 18 (true positives) and missed 52 (false negatives); among the six eyes classified as normal by the specialist, the AI flagged four (false positives) and correctly classified two (true negatives). The system demonstrated sensitivity of 25.7% (95%CI 16.9–37.0), specificity of 33.3% (95%CI 9.7–70.0), PPV of 81.8% (95%CI 61.5–92.7), NPV of 3.7% (95%CI 1.0–12.5), and overall accuracy of 26.3% (95%CI 17.7–37.2). These metrics reflect detection of any ocular abnormality rather than diabetic retinopathy specifically. Despite the very low sensitivity—indicating that the majority of abnormal cases were missed—the PPV of 81.8% suggests that when the system flags an abnormality, the finding is likely genuine, although the wide CI for specificity (based on only six normal eyes) indicates this estimate is imprecise.

## DISCUSSION

This study demonstrated that ocular screening using the Eyer^®^ portable fundus camera by non-ophthalmologist professionals is operationally feasible in an LTCF setting. Images were obtained in all recruited participants without transfer, following brief ophthalmologist-led training, corroborating prior studies validating portable cameras in limited-resource environments^6,7^.

The very high prevalence of ocular abnormalities (92.1%) was the most striking finding, with glaucoma suspicion, cataract, atrophic chorioretinal changes, and AMD predominating—conditions unrelated to diabetic retinopathy. This has relevant clinical implications: a substantial proportion of residents may benefit from ophthalmological evaluation regardless of diabetes diagnosis, and LTCF screening strategies should address a broader spectrum of ocular disease. Media opacities compromised image quality in some cases, a challenge inherent to elderly populations and likely accentuated by non-mydriatic acquisition; systematic mydriasis and integration with cataract detection programs may mitigate this in future protocols.

No diabetic retinopathy was identified among gradable eyes. Among the 15 participants with confirmed diabetes, this may be consistent with recent diagnosis and/or adequate metabolic control, in which established retinopathy would not yet be expected. This interpretation remains tentative, as glycated hemoglobin and diabetes duration were unavailable; the small diabetic subgroup, self-reported diabetes status, and media opacities limiting fundus assessment may also account for the absence of detected retinopathy.

EyerMaps^®^ sensitivity (25.7%) was substantially lower than the 80–96% reported in diabetic retinopathy screening validation studies^8^. This reflects a key methodological difference: validation studies were conducted in selected diabetic populations with a focus on referable diabetic retinopathy, whereas we compared AI performance against all ocular findings, including glaucoma, AMD, and chorioretinal atrophies for which the algorithm was not trained. These metrics should therefore be read as the algorithm's performance for detection of any abnormality, not as a validation for its intended indication; rather, they illustrate the risk of applying a disease-specific algorithm as a general screening tool in a population with a high non-diabetic pathology burden. Nevertheless, the PPV of 81.8% preserves its utility as a prioritization tool.

From a public health perspective, these findings suggest that LTCF screening programs should be designed around the broad spectrum of age-related ocular disease rather than diabetic retinopathy alone, paired with structured referral pathways for cataract and glaucoma. Portable, non-mydriatic imaging with remote interpretation is compatible with existing Unified Health System (SUS) primary-care and telehealth workflows, although formal cost and resource evaluation is needed.

Limitations include the absence of digitized records (precluding HbA1c and diabetes duration data), the small sample (38 participants, 76 eyes) with correspondingly wide CIs limiting the precision of diagnostic estimates and generalizability, recruitment failures introducing potential selection bias, self-reported diabetes status, and the methodological limitation of comparing AI performance to all ocular findings rather than diabetic retinopathy alone. Findings may also differ across Brazilian regions and LTCF profiles, and socioeconomic factors likely influence screening uptake. Future studies should include stratified analyses by finding type, structured recruitment workflows, protocols for non-gradable images, and image-quality strategies for media opacity.

## CONCLUSION

The Eyer^®^ portable fundus camera demonstrated operational feasibility for ocular screening by trained non-ophthalmologist professionals at an LTCF, without requiring patient transfer. The high prevalence of ocular abnormalities—predominantly unrelated to diabetic retinopathy—and the resultant compromise of AI performance highlight that LTCF screening strategies must go beyond diabetic retinopathy and be supported by organized referral pathways to specialized care. Portable fundus photography with remote interpretation is a promising tool for broadening ocular diagnostic access in vulnerable populations when implemented within structured, context-aware protocols.

## Data Availability

The datasets generated and/or analyzed during the current study are available from the corresponding author upon reasonable request.
